# One health risk assessment of heavy-metal contamination in Nile tilapia (Oreochromis niloticus) from contrasting aquaculture systems in Egypt

**DOI:** 10.1016/j.toxrep.2026.102331

**Published:** 2026-08-16

**Authors:** Mohamed Farahat Abdelmawla, Ahmed M.D. Elshafei, Alam Eldeen Farouk, Mohamed A. Al-Zahaby, Ibrahim Shaker

**Affiliations:** a University of Innsbruck, Innsbruck 6020, Austria; b Central Laboratory for Aquaculture Research (CLAR), Agricultural Research Centre (ARC), Abbassa, Abou-Hammad, Sharkia, Egypt

**Keywords:** One Health, Heavy metals, Bioaccumulation, Nile tilapia, Human health risk, THQ, Hazard Index, Drainage water, BAF

## Abstract

In water-scarce Egypt, aquaculture relies on agricultural drainage water, raising health concerns. Water and Nile tilapia were sampled seasonally from four sites (drainage versus Nile freshwater). Lead (Pb), cadmium (Cd), chromium (Cr), and mercury (Hg) were measured in water and fish tissues by ICP-MS and FAAS. Bioaccumulation factors (BAFs) and health risks (EDI, THQ, HI) were calculated. Kitchener Drain showed severe contamination, with Pb, Cd, Cr, and Hg exceeding WHO limits 5–54-fold. Bioaccumulation followed liver > kidney > muscle, with Pb reaching 4.90 mg/kg in summer liver. Muscle remained within FAO/WHO limits. BAFs showed metal transfer even at low ambient levels. All THQ values were < 1. The HI at Kitchener Drain (0.36) was 3.6-fold higher than reference sites (HI=0.10), peaking in summer. Correlations between water, tissue, and human health risk were strong (r > 0.85, p < 0.001). Although edible muscle poses no immediate risk, narrow Pb safety margins (0.28 mg/kg vs 0.3 mg/kg) and higher cumulative risk highlight the need for monitoring and water treatment. This One Health assessment supports sustainable aquaculture management in Egypt.

## Highlights

• Drainage-fed aquaculture shows severe heavy metal pollution (Pb, Cd, Cr, Hg).
• Heavy metal bioaccumulation follows: liver > kidney > muscle in Nile tilapia.
• Muscle Pb (0.28 mg/kg) approaches FAO/WHO limit (0.3 mg/kg).
• Summer Hazard Index (HI = 0.36) is 3.6 × higher than at reference sites.
• One Health framework links water quality → fish contamination → human risk.

## 1 Introduction

### 1.1 The one health concept in aquaculture

The One Health approach recognises that human, animal, and environmental health are deeply interconnected. This interdisciplinary concept has gained global recognition for addressing complex health challenges that transcend traditional disciplinary boundaries[1]. In aquaculture, One Health offers a framework for understanding how environmental pollution affects farmed fish and, ultimately, human consumers through the food chain.

### 1.2 Aquaculture in Egypt: a one health challenge

Egypt is Africa's largest aquaculture producer, with an annual output of approximately 1.6 million tonnes, representing about 75% of the country's total fish output [2], [3]. A detailed analysis of Egypt’s water resources is presented by Nour El Din [4]. FAO [5] provides an overview of the Egyptian aquaculture sector. Nile tilapia (Oreochromis niloticus) accounts for around 80% of freshwater-farmed fish and is a crucial source of affordable animal protein for the Egyptian population [6].

However, Egypt faces severe water shortages, with the Nile River supplying about 55.5 billion cubic metres annually, of which 85% is used for irrigation [4]. This limited supply has led to the widespread reuse of agricultural drainage water in aquaculture, particularly in the Nile Delta [7], [8]. The Kitchener Drain, one of Egypt's largest agricultural drains, runs for approximately 69 km through the Nile Delta, receiving agricultural, industrial, domestic, and municipal waste [9]. Despite concerns about its poor water quality, it remains a vital resource for aquaculture in the Kafr Elsheikh Governorate. This presents a classic One Health dilemma: balancing economic necessity against potential health risks.

### 1.3 Heavy metal pollution: a one health threat

Heavy metals such as lead (Pb), cadmium (Cd), chromium (Cr), and mercury (Hg) pose persistent environmental risks due to their toxicity, non-biodegradability, and tendency to bioaccumulate in aquatic organisms [10], [11]. These pollutants enter water bodies through agricultural runoff, industrial effluents, and untreated sewage [12], [13], [14].

Tissue‑specific accumulation of heavy metals, with higher concentrations in the liver and kidney than in muscle, has been observed in various fish species [15], [16]. Elevated metal levels in fish from contaminated rivers have been documented by Brumbaugh et al. [17]. Baseline heavy metal concentrations across different fish species have also been analysed by Yilmaz et al. [18]. The use of fish as bioindicators of aquatic ecosystem health is reviewed by Stankovic and Stankovic [19]. The negative effects of ammonia on growth and physiological performance in Nile tilapia have been demonstrated by Abdel-Tawwab et al., [20].

**Table:** 

Domain	Impact	Reference
Environmental Health	Water quality degradation. ecosystem disruption	[21]
Animal Health	Bioaccumulation in fish tissues. physiological stress	[22], [23]
Human Health	Dietary exposure through fish consumption	[24]; WHO. 2011

From a One Health perspective. heavy metal contamination affects:

### 1.4 Biomarkers as one health tools

Biomarkers provide measurable indicators of exposure and effect across the One Health continuum:

**Table:** 

Biomarker	Domain	Significance
Heavy metals in water	Environmental	Contamination level
Heavy metals in fish tissues	Animal	Bioaccumulation, exposure
Metal concentrations in muscle	Human	Dietary exposure
THQ/HI indices	Human	Health risk assessment

### 1.5 Research gaps

While previous studies have examined metal pollution in Egyptian waters or investigated fish physiology, no research has integrated all three One Health domains into a comprehensive assessment. Specifically, there is a lack of:

1. Quantitative links between environmental contamination and Fisch physiological responses
2. Integration of fish biomarker data with human health risk assessment
3. Seasonal analysis of One Health risks
4. Evidence-based policy recommendations within a One Health framework

### 1.6 Study objectives (one health framework)

This study aims to fill these gaps through a comprehensive investigation designed to:

1. **Environmental health domain:** Quantify seasonal heavy metal concentrations (Pb, Cd, Cr, Hg) in water from contrasting aquaculture systems.
2. **Animal health domain:** *"Assess metal bioaccumulation in fish tissues (liver, kidney, muscle)."*
3. **Human health domain:** Assess dietary exposure and health risks for consumers using Estimated Daily Intake (EDI), Target Hazard Quotient (THQ), and Hazard Index (HI).
4. **Integration domain:** Establish correlations between environmental. animal. and human health parameters to validate the One Health approach.
5. **Policy domain:** Develop evidence-based recommendations for sustainable aquaculture management within a One Health framework.

### 1.7 Scientific contribution

This work offers the first comprehensive One Health evaluation of heavy metal contamination in Egyptian aquaculture. combining environmental monitoring. fish physiology. and human health risk assessment. The results will guide policy decisions and create a framework for sustainable aquaculture management in water-scarce areas

## 2 Materials and methods

### 2.1 Study sites

The study was conducted at four locations representing different aquaculture water sources in Egypt:

**Table:** 

Site	Water Source	Characteristics	Coordinates
Kitchener Drain	Agricultural drainage water	Heavily polluted. receives industrial. domestic. and agricultural effluents	31°05' N. 30°55' E
Kitchener Fish Farm	Kitchener Drain water	Private farm using drainage water for aquaculture	31°07' N. 30°58' E
Ismailia Canal	Nile freshwater	Relatively clean. fed directly by the Nile	30°35' N. 32°15' E
Abbassa Fish Farm	Nile freshwater	Research farm (World Fish Center) using clean freshwater	30°45' N. 31°35' E

These sites were selected to compare drainage-affected systems and clean freshwater environments. Geographic coordinates were recorded using GPS, and all sites were sampled seasonally from autumn to summer [9].

### 2.2 Water sampling and physico-chemical analysis

Water samples were collected seasonally using pre-cleaned, sterilised polyethene bottles at a depth of 30 cm from each location. Key physico-chemical parameters were measured using portable multiparameter meters (Hach, USA) in accordance with standard APHA [25] protocols.

All measurements were performed in triplicate [2], [25]

**Table:** 

Parameter	Method/Instrument	Reference
Temperature	Portable meter (Hach HQ40d)	APHA 2550 B
pH	Portable pH meter (Hach pH meter)	APHA 4500-H⁺ B
Dissolved Oxygen (DO)	DO meter (Hach LDO)	APHA 4500-O G
Electrical Conductivity (EC)	Conductivity meter (Hach CDC401)	APHA 2510 B
Total Dissolved Solids (TDS)	Calculated from EC	APHA 2540 C
Ammonia (NH₃)	Spectrophotometric (Nesslerization)	[26]
Nitrite (NO₂⁻)	Spectrophotometric (Diazotization)	APHA 4500-NO₂⁻ B
Nitrate (NO₃⁻)	Spectrophotometric (Cadmium reduction)	APHA 4500-NO₃⁻ E

All measurements were performed in triplicate ( [25])

### 2.3 Heavy metal analysis in water

Water samples for metal analysis were acidified with concentrated nitric acid (HNO₃, 65%, Merck, Germany) to a pH below 2 and stored at 4°C until analysis. Concentrations of lead (Pb), cadmium (Cd), chromium (Cr), and mercury (Hg) were determined by Inductively Coupled Plasma Mass Spectrometry (ICP-MS, Agilent 7900) following standard procedures [25]. Calibration curves were prepared from certified standards (Merck, Germany). Detection limits were: Pb, 0.01 µg/L; Cd, 0.005 µg/L; Cr, 0.02 µg/L; Hg, 0.001 µg/L.

### 2.4 Fish sampling

Nile tilapia (Oreochromis niloticus) specimens were collected seasonally (autumn, winter, spring, summer) from all four sites. At each site and season, ten fish of similar size (average weight: 120–150 g, average length: 18–22 cm) were randomly caught by netting to minimise the influence of size on metal accumulation (n = 10 per site per season, total n = 160). Fish were transported to the laboratory in ice boxes, where they were measured for:

• Total length (cm) using ichthyometer
• Body weight (g) using digital balance (Sartorius, Germany)

*"Fish were obtained from the respective aquaculture sites (Kitchener Drain, Kitchener Fish Farm, Ismailia Canal, and Abbassa Fish Farm) as part of routine sampling conducted by the Central Laboratory for Aquaculture Research (CLAR)."*

### 2.5 Tissue sampling and heavy metal analysis

− Fish were dissected with stainless-steel tools to collect liver, kidney, and dorsal muscle tissue. Tissue samples were rinsed with distilled water, placed in polyethylene bags, and stored at −20°C until analysis.
− For heavy metal analysis in fish tissues, the following procedure was used [27], [28]: [dann die 10 Schritte wie bei dir]
− Quality assurance included the analysis of certified reference material (DORM−4, fish protein, National Research Council Canada) and reagent blanks. Recovery rates ranged from 92% to 106% for all metals.

### 2.6 Calculation

Concentration (mg/kg wet weight) = (A-B) × V / M

where A = concentration of test solution (µg/ml). B = concentration of blank solution (µg/ml). V = volume of test solution (ml). M = wet weight of tissue (g).

Quality assurance included the analysis of certified reference material (DORM−4, fish protein, National Research Council Canada) and reagent blanks. Recovery rates ranged from 92% to 106% for all metals.

### 2.7 Bioaccumulation factor (BAF)

The bioaccumulation factor was calculated to assess the extent of heavy metal transfer from water to fish tissues.

**BAF = C_tissue / C_water**

where C_tissue is the metal concentration in fish tissue (mg/kg wet weight), and C_water is the metal concentration in water (mg/L) [11], [29].

### 2.8 Human health domain: risk assessment

#### 2.8.1 Estimated daily intake (EDI)

The estimated daily intake of heavy metals via fish consumption was determined using:

**EDI = (C_muscle × IR) / BW**

where:

• C_muscle = metal concentration in muscle tissue (mg/kg wet weight)
• IR = daily ingestion rate (kg/person/day) - Egyptian average: 0.115 kg/person/day [2]
• BW = average body weight (kg) - adult: 70 kg (US EPA. 2011)

#### 2.8.2 Target hazard quotient (THQ)

The THQ estimates the non-carcinogenic health risk from individual metals:

**THQ = (EDI × EF × ED) / (RfD × AT × 365)**

where:

• EF = exposure frequency (365 days/year)
• ED = exposure duration (70 years, average lifetime)
• RfD = oral reference dose (mg/kg/day) (US EPA. 2021)
• AT = averaging time (70 years × 365 days)

**RfD values (US EPA. 2021):**

• Pb: 3.5 × 10⁻³ mg/kg/day
• Cd: 1.0 × 10⁻³ mg/kg/day
• Cr: 3.0 × 10⁻³ mg/kg/day
• Hg: 3.0 × 10⁻⁴ mg/kg/day

**THQ < 1:** No significant health risk

**THQ > 1:** Potential health risk

#### 2.8.3 Hazard index (HI)

The HI represents the cumulative non-carcinogenic risk from multiple metals:

**HI = Σ THQ (Pb + Cd + Cr + Hg)**

**HI < 1:** No significant cumulative risk

**HI > 1:** Potential cumulative health risk

### 2.9 Statistical analysis

All data were tested for normality (Shapiro-Wilk test) and homogeneity of variance (Levene's test). Data were analysed in SPSS v25.0 (IBM, USA). A two-way ANOVA was used to assess significant differences among sites and seasons, with post hoc comparisons conducted using the Scheffé test. Pearson correlation analysis was performed to examine relationships between metal concentrations in water and tissues. Significance was set at *p* < 0.05. Results were expressed as mean ± standard deviation (SD).

### 2.10 Ethical statement

"The study was conducted in accordance with the guidelines of the Central Laboratory for Aquaculture Research (CLAR), Abbassa, Egypt. The experimental protocol involving Nile tilapia (Oreochromis niloticus) was approved by the **Institutional Animal Care and Use Committee of the Central Laboratory for Aquaculture Research (CLAR-IACUC)**. All efforts were made to minimise animal suffering and to reduce the number of fish used."

## 3 Results

### 3.1 Water quality parameters

**Fig. 1:**
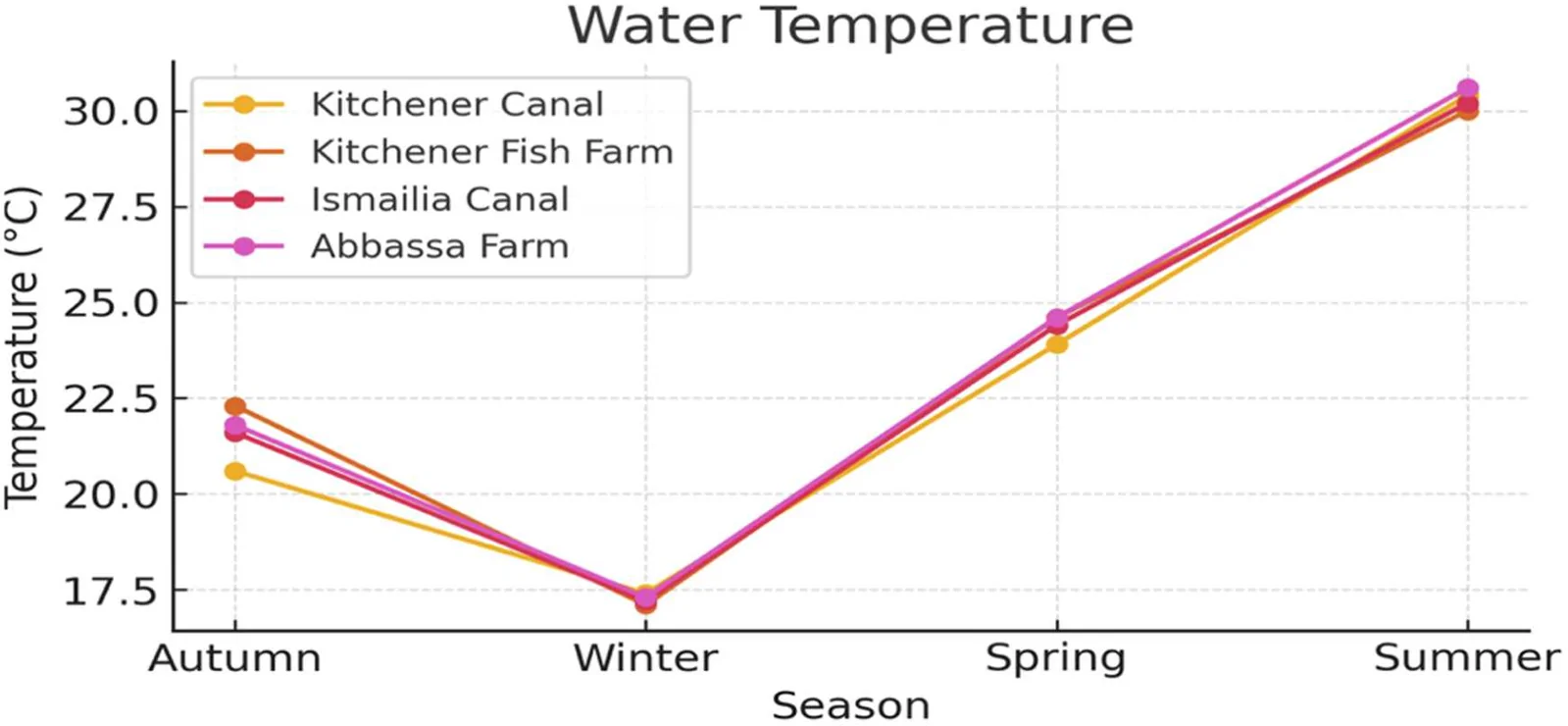
Seasonal water temperature (°C) at four aquaculture sites in Egypt (autumn - summer). Values are mean ± SD (n = 3 per site per season). Seasonal differences were statistically significant (p < 0.001).

**Fig. 2:**
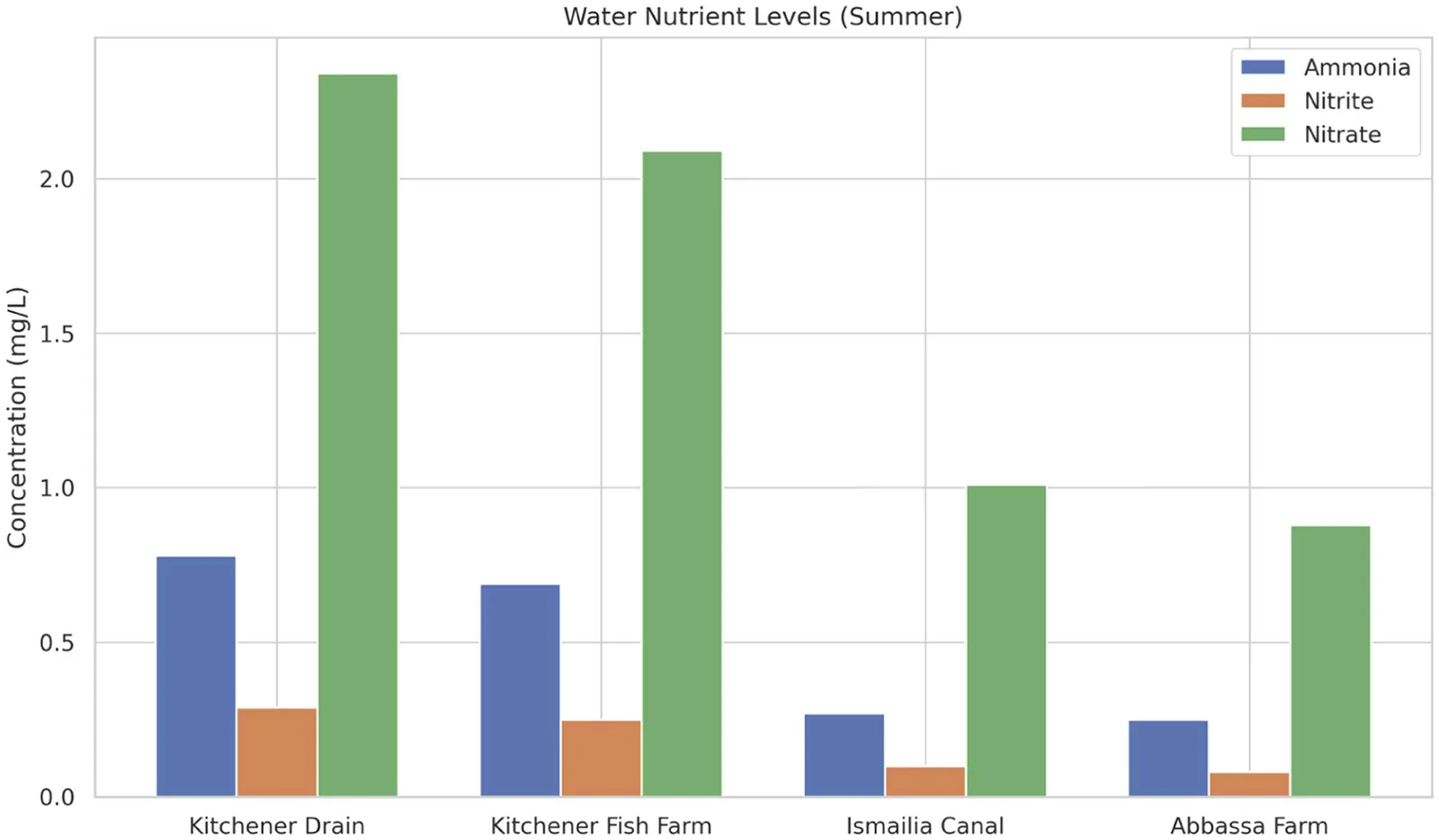
Seasonal water nutrient levels (ammonia, nitrite, nitrate) at the four study sites during summer.

**Fig. 3:**
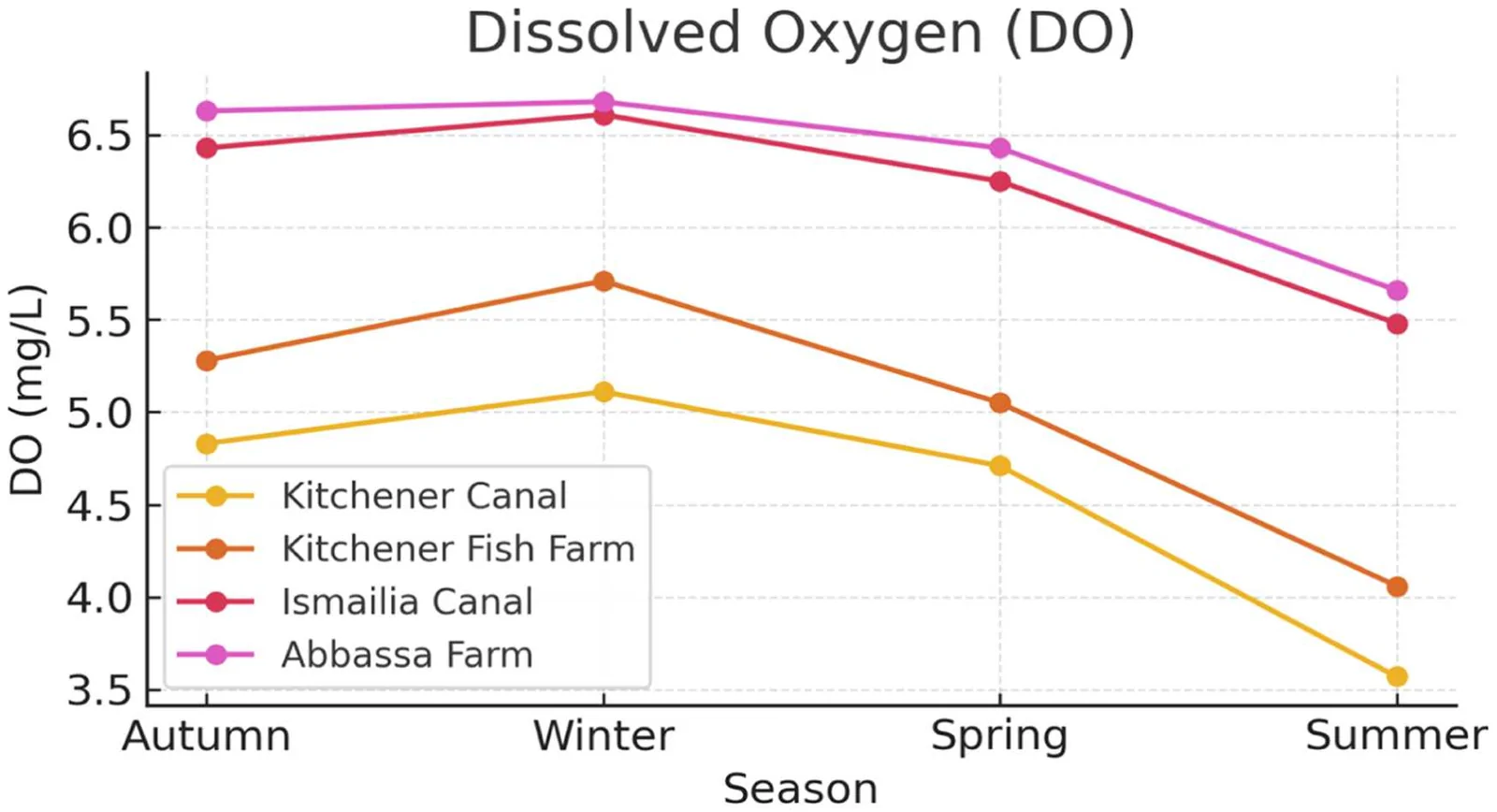
Seasonal dissolved oxygen (DO) concentrations (mg/L) at four aquaculture sites. The dashed red line indicates the hypoxic threshold (4 mg/L). Site and seasonal differences were significant (p < 0.001).

**Fig. 4:**
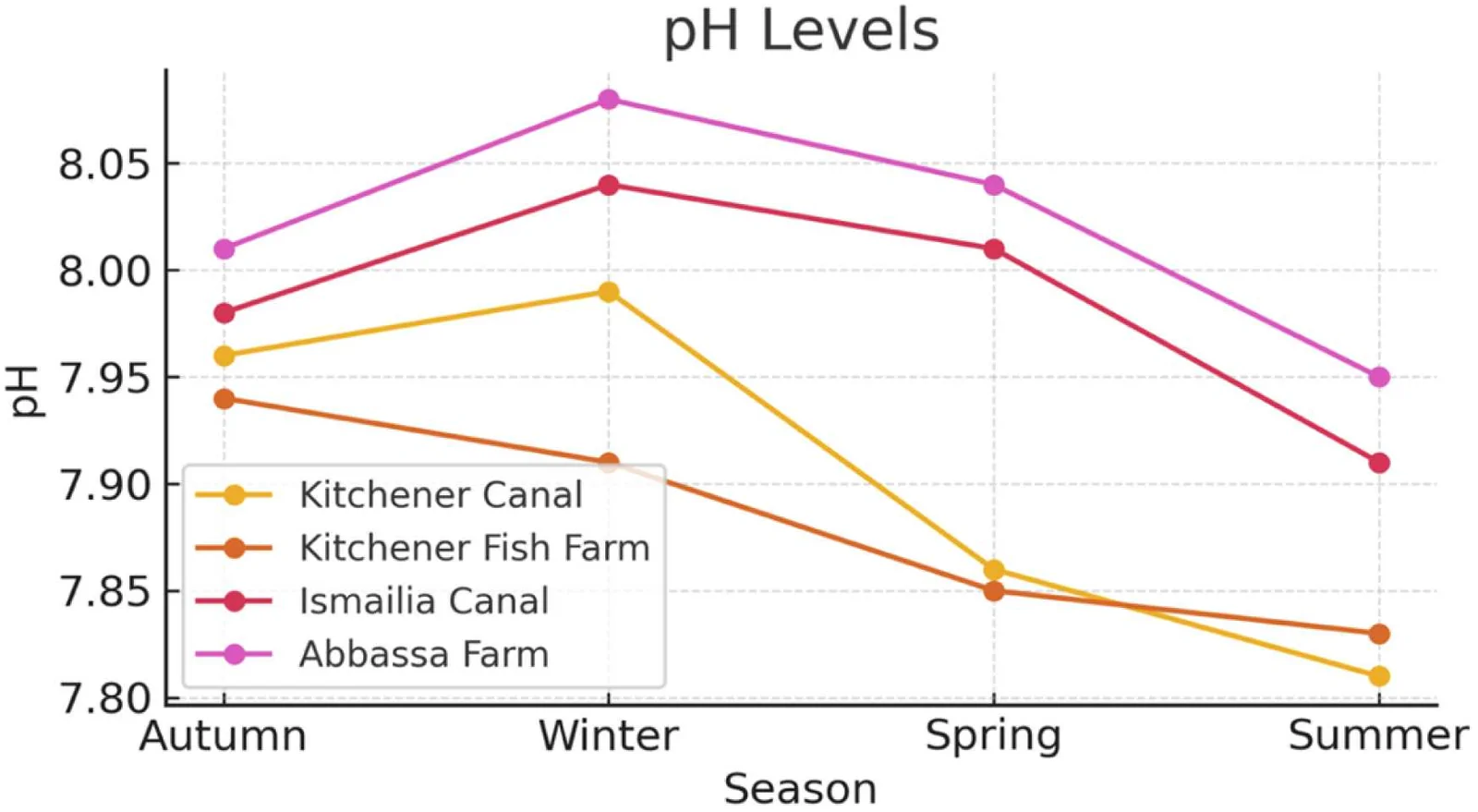
Seasonal pH values at four aquaculture sites. No significant differences were observed between sites (p > 0.05).

**Fig. 5:**
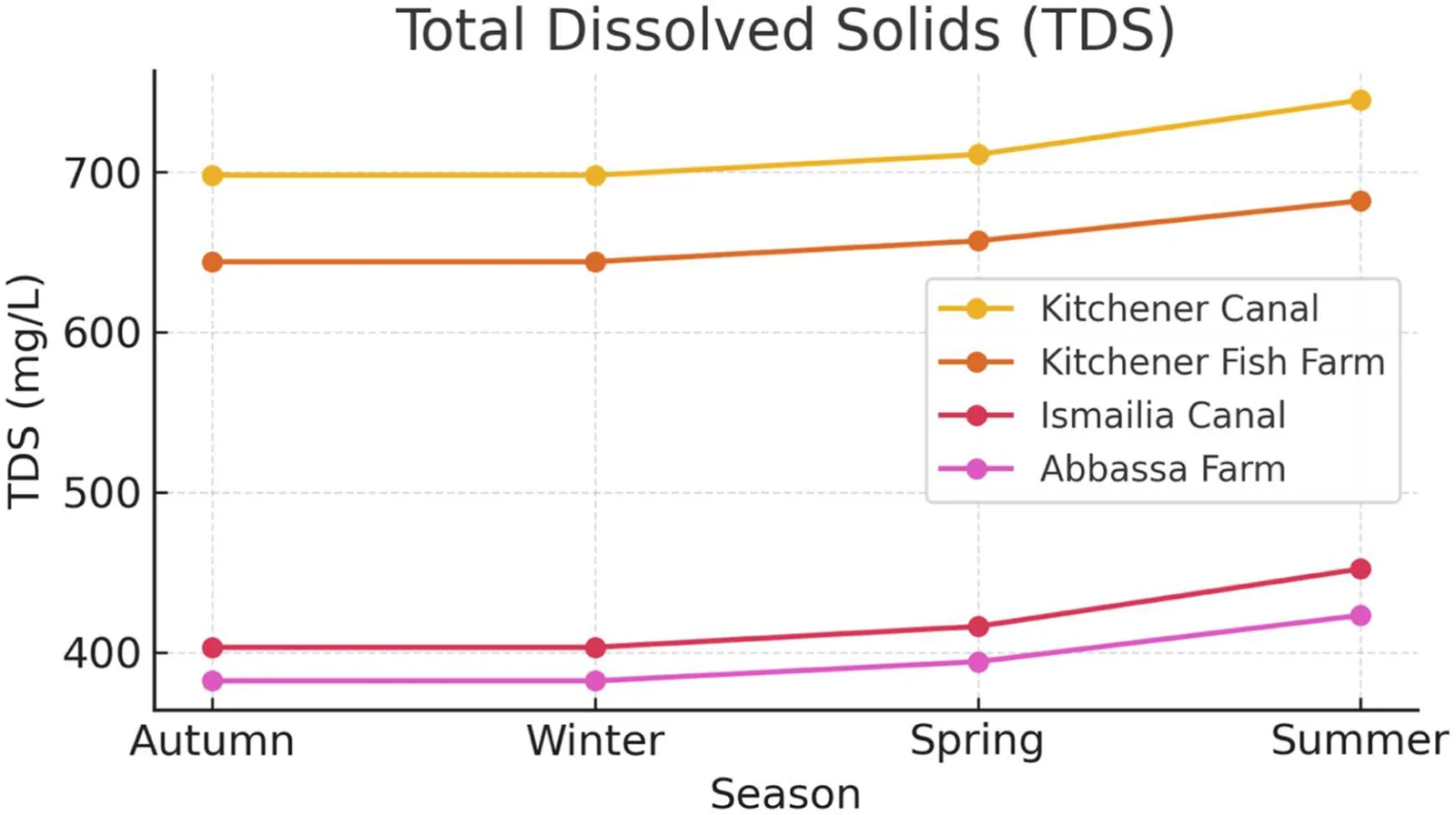
Seasonal total dissolved solids (TDS) concentrations (mg/L) at four aquaculture sites. Site and seasonal differences were significant (p < 0.001).

**Fig. 6:**
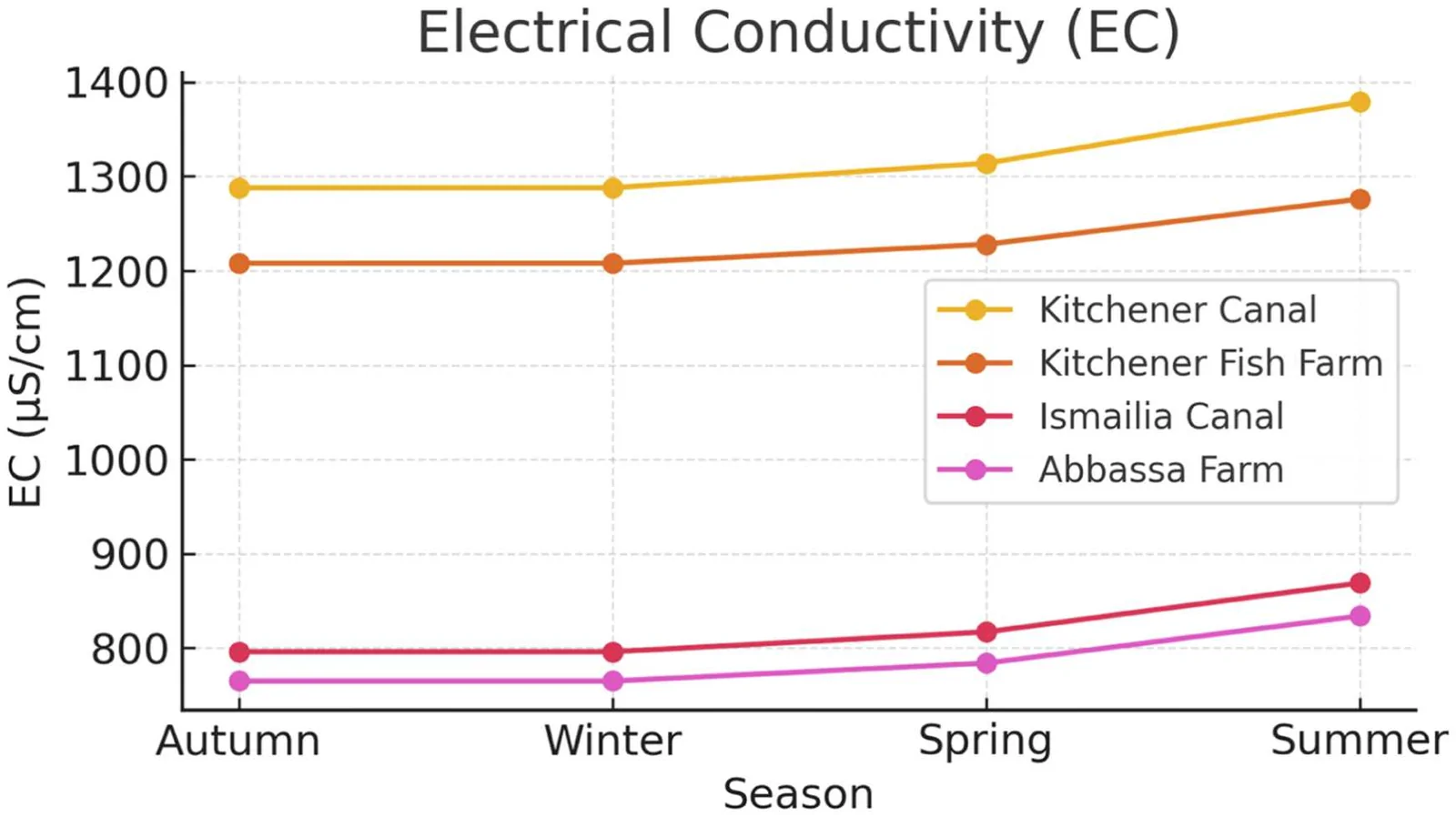
Seasonal electrical conductivity (EC, µS/cm) at four aquaculture sites. Site and seasonal differences were significant (p < 0.001).

The physico-chemical parameters showed notable seasonal and spatial variation across the study sites. Temperature and nutrient concentrations were significantly higher in summer, particularly at the Kitchener Drain and its associated fish farm **(**Fig. 1**)**. Ammonia and nitrate levels rose in spring and summer, reaching peak values of 0.78 mg/L and 2.34 mg/L, respectively, at the Kitchener sites (Table 1). * Indicating substantial agricultural runoff and organic waste inputs **(**Fig. 2**)**. Dissolved oxygen (DO) levels fell to 3.57 mg/L in the Kitchener Drain during summer, signalling near-hypoxic conditions that are unsuitable for the development of healthy fish **(**Fig. 3**)**. The pH values remained relatively stable across all sites and seasons, with no significant differences observed **(**Fig. 4**)**. Total dissolved solids (TDS) and electrical conductivity (EC) were significantly elevated at the Kitchener sites, particularly during summer, reflecting higher salinity and ion concentrations **(**Fig. 5, Fig. 6**)**. In contrast, the Ismailia and Abbassa sites maintained DO values above 5.5 mg/L and showed consistently lower TDS and EC throughout the year are shown in (Table 2)

**Table 1:** Mean water temperature (°C), ammonia (NH₃), nitrite (NO₂⁻), and nitrate (NO₃⁻) concentrations (mg/L) at four aquaculture sites across seasons. Values are mean ± SD (n = 3 per site per season).

Season	Site	Temperature (°C)	Ammonia (NH₃) (mg/L)	Nitrite (NO₂⁻) (mg/L)	Nitrate (NO₃⁻) (mg/L)
Autumn	Kitchener Drain	20.6 ± 0.95	0.58 ± 0.03	0.19 ± 0.02	1.84 ± 0.07
	Kitchener Fish Farm	22.3 ± 0.83	0.48 ± 0.02	0.15 ± 0.01	1.63 ± 0.05
	Ismailia Canal	21.6 ± 0.35	0.21 ± 0.01	0.07 ± 0.01	0.83 ± 0.03
	Abbassa Farm	21.8 ± 0.29	0.19 ± 0.01	0.06 ± 0.01	0.72 ± 0.02
Winter	Kitchener Drain	17.4 ± 0.48	0.49 ± 0.03	0.17 ± 0.02	1.61 ± 0.06
	Kitchener Fish Farm	17.1 ± 0.25	0.40 ± 0.02	0.12 ± 0.01	1.39 ± 0.04
	Ismailia Canal	17.2 ± 0.30	0.18 ± 0.01	0.05 ± 0.01	0.68 ± 0.03
	Abbassa Farm	17.3 ± 0.29	0.17 ± 0.01	0.05 ± 0.01	0.64 ± 0.02
Spring	Kitchener Drain	23.9 ± 0.48	0.67 ± 0.04	0.24 ± 0.02	2.11 ± 0.09
	Kitchener Fish Farm	24.6 ± 0.48	0.58 ± 0.03	0.21 ± 0.02	1.89 ± 0.08
	Ismailia Canal	24.4 ± 0.31	0.24 ± 0.01	0.08 ± 0.01	0.93 ± 0.04
	Abbassa Farm	24.6 ± 0.25	0.22 ± 0.01	0.07 ± 0.01	0.82 ± 0.03
Summer	Kitchener Drain	30.4 ± 0.48	0.78 ± 0.05	0.29 ± 0.03	2.34 ± 0.11
	Kitchener Fish Farm	30.0 ± 0.41	0.69 ± 0.04	0.25 ± 0.02	2.09 ± 0.10
	Ismailia Canal	30.2 ± 0.38	0.27 ± 0.01	0.10 ± 0.01	1.01 ± 0.05
	Abbassa Farm	30.6 ± 0.48	0.25 ± 0.01	0.08 ± 0.01	0.88 ± 0.04

Two-way ANOVA results: Site effect: p < 0.001 for all parameters; Season effect: p < 0.001 for all parameters.

**Table 2:** Dissolved oxygen (DO, mg/L), pH, total dissolved solids (TDS, mg/L), and electrical conductivity (EC, µS/cm) at four aquaculture sites across seasons. Values are mean ± SD (n = 3 per site per season).

Season	Site	DO (mg/L)	pH	TDS (mg/L)	EC (µS/cm)
Autumn	Kitchener Drain	4.83 ± 0.06	7.96 ± 0.04	698 ± 2.65	1288 ± 9.40
	Kitchener Fish Farm	5.28 ± 0.05	7.94 ± 0.07	644 ± 2.94	1208 ± 11.85
	Ismailia Canal	6.43 ± 0.08	7.98 ± 0.06	403 ± 1.96	796 ± 7.20
	Abbassa Farm	6.63 ± 0.06	8.01 ± 0.03	382 ± 1.41	765 ± 6.38
Winter	Kitchener Drain	5.11 ± 0.07	7.99 ± 0.05	698 ± 2.65	1288 ± 9.40
	Kitchener Fish Farm	5.71 ± 0.08	7.91 ± 0.06	644 ± 2.94	1208 ± 11.85
	Ismailia Canal	6.61 ± 0.05	8.04 ± 0.04	403 ± 1.96	796 ± 7.20
	Abbassa Farm	6.68 ± 0.07	8.08 ± 0.04	382 ± 1.41	765 ± 6.38
Spring	Kitchener Drain	4.71 ± 0.08	7.86 ± 0.05	711 ± 3.06	1314 ± 8.40
	Kitchener Fish Farm	5.05 ± 0.06	7.85 ± 0.03	657 ± 2.95	1228 ± 10.88
	Ismailia Canal	6.25 ± 0.06	8.01 ± 0.04	416 ± 2.33	817 ± 6.74
	Abbassa Farm	6.43 ± 0.05	8.04 ± 0.06	394 ± 1.57	784 ± 5.85
Summer	Kitchener Drain	3.57 ± 0.05	7.81 ± 0.07	745 ± 3.55	1379 ± 8.66
	Kitchener Fish Farm	4.06 ± 0.05	7.83 ± 0.06	682 ± 3.26	1276 ± 10.59
	Ismailia Canal	5.48 ± 0.06	7.91 ± 0.05	452 ± 2.15	869 ± 7.50
	Abbassa Farm	5.66 ± 0.07	7.95 ± 0.04	423 ± 1.48	834 ± 6.23

Two-way ANOVA results: Site effect: p < 0.001 for DO. TDS. EC; p > 0.05 for pH; Season effect: p < 0.001 for DO. TDS. EC. Bold values indicate critical levels (DO < 4 mg/L) and peak TDS/EC.

### 3.2 Heavy metals in water

**Fig. 7:**
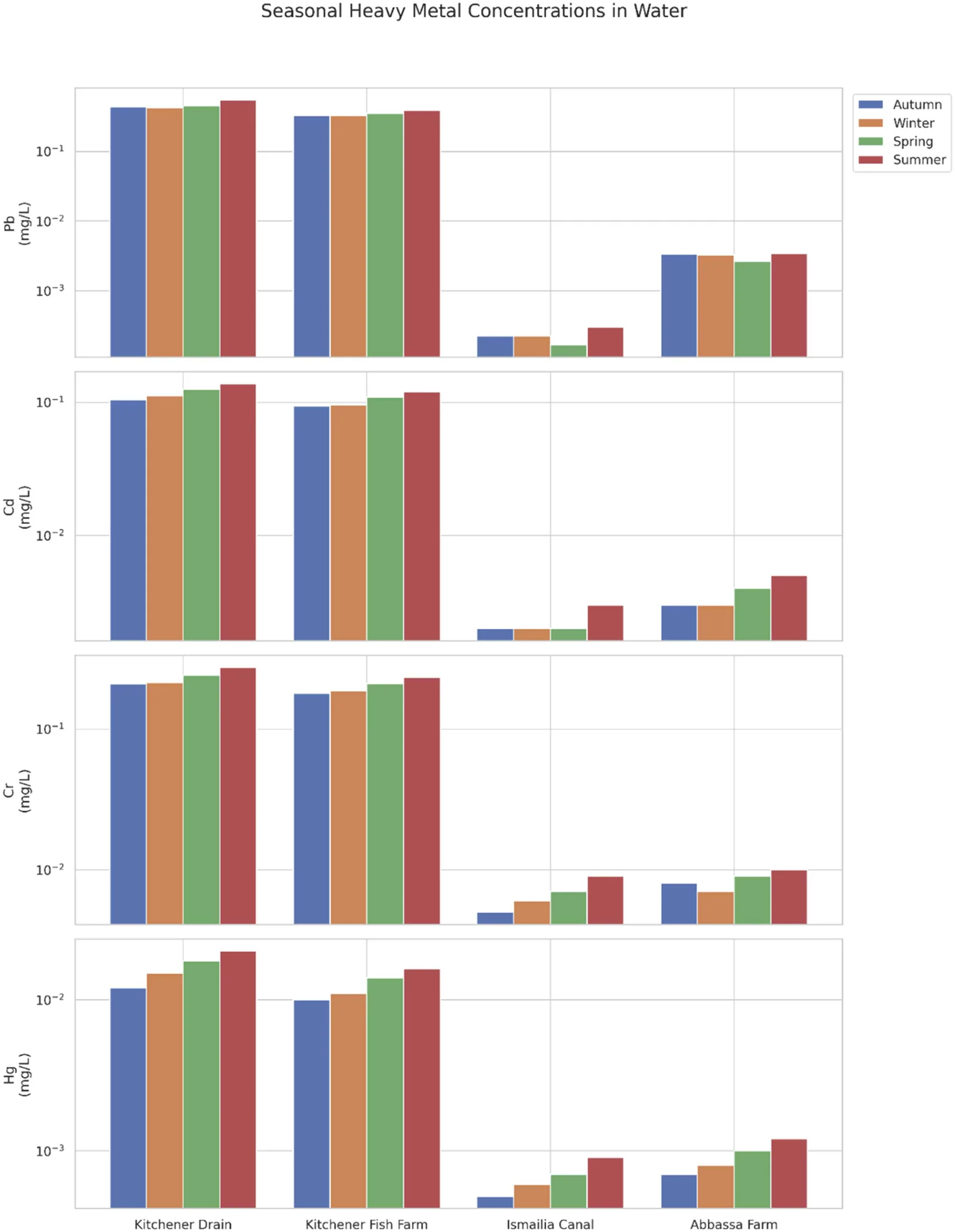
Heavy metal concentrations (Pb, Cd, Cr, Hg) in water at the four study sites across all seasons.

The concentrations of heavy metals in water followed the order: Pb > Cd > Cr > Hg, with the highest levels consistently recorded at the Kitchener Drain, especially during summer. Notably, lead, cadmium, and chromium reached 0.54 mg/L, 0.14 mg/L, and 0.28 mg/L, respectively, at Kitchener sites, all exceeding international safety guidelines [30]. The Ismailia Canal and Abbassa showed significantly lower concentrations within permissible limits (Table 3). The seasonal dynamics of heavy metal concentrations in water across all four sites are illustrated in Fig. 7**.**

**Table 3:** Seasonal concentrations of heavy metals (mg/L) in water at the four study sites. Values are presented as mean ± SD (n = 3). WHO limits: Pb 0.01 mg/L, Cd 0.005 mg/L, Cr 0.05 mg/L, Hg 0.001 mg/L.

Season	Site	Pb (mg/L)	Cd (mg/L)	Cr (mg/L)	Hg (mg/L)
Autumn	Kitchener Drain	0.4355 ± 0.021	0.105 ± 0.008	0.210 ± 0.015	0.012 ± 0.001
	Kitchener Fish Farm	0.3283 ± 0.018	0.094 ± 0.007	0.180 ± 0.012	0.010 ± 0.001
	Ismailia Canal	0.000225 ± 0.00002	0.002 ± 0.0002	0.005 ± 0.0004	0.0005 ± 0.00005
	Abbassa Farm	0.00335 ± 0.0003	0.003 ± 0.0003	0.008 ± 0.0007	0.0007 ± 0.00006
Winter	Kitchener Drain	0.4248 ± 0.020	0.112 ± 0.009	0.215 ± 0.016	0.015 ± 0.001
	Kitchener Fish Farm	0.3243 ± 0.017	0.096 ± 0.008	0.188 ± 0.013	0.011 ± 0.001
	Ismailia Canal	0.000225 ± 0.00002	0.002 ± 0.0002	0.006 ± 0.0005	0.0006 ± 0.00006
	Abbassa Farm	0.00328 ± 0.0003	0.003 ± 0.0003	0.007 ± 0.0006	0.0008 ± 0.00007
Spring	Kitchener Drain	0.4503 ± 0.022	0.126 ± 0.010	0.242 ± 0.018	0.018 ± 0.002
	Kitchener Fish Farm	0.3533 ± 0.019	0.110 ± 0.009	0.212 ± 0.015	0.014 ± 0.001
	Ismailia Canal	0.000168 ± 0.00002	0.002 ± 0.0002	0.007 ± 0.0006	0.0007 ± 0.00007
	Abbassa Farm	0.00265 ± 0.0002	0.004 ± 0.0004	0.009 ± 0.0008	0.0010 ± 0.00009
Summer	Kitchener Drain	0.5435 ± 0.025	0.138 ± 0.011	0.275 ± 0.020	0.021 ± 0.002
	Kitchener Fish Farm	0.3900 ± 0.020	0.120 ± 0.010	0.235 ± 0.017	0.016 ± 0.002
	Ismailia Canal	0.000300 ± 0.00003	0.003 ± 0.0003	0.009 ± 0.0008	0.0009 ± 0.00008
	Abbassa Farm	0.00343 ± 0.0003	0.005 ± 0.0005	0.010 ± 0.0009	0.0012 ± 0.0001

### 3.3 Heavy metal accumulation in fish tissues

#### 3.3.1 Liver tissue

**Fig. 8:**
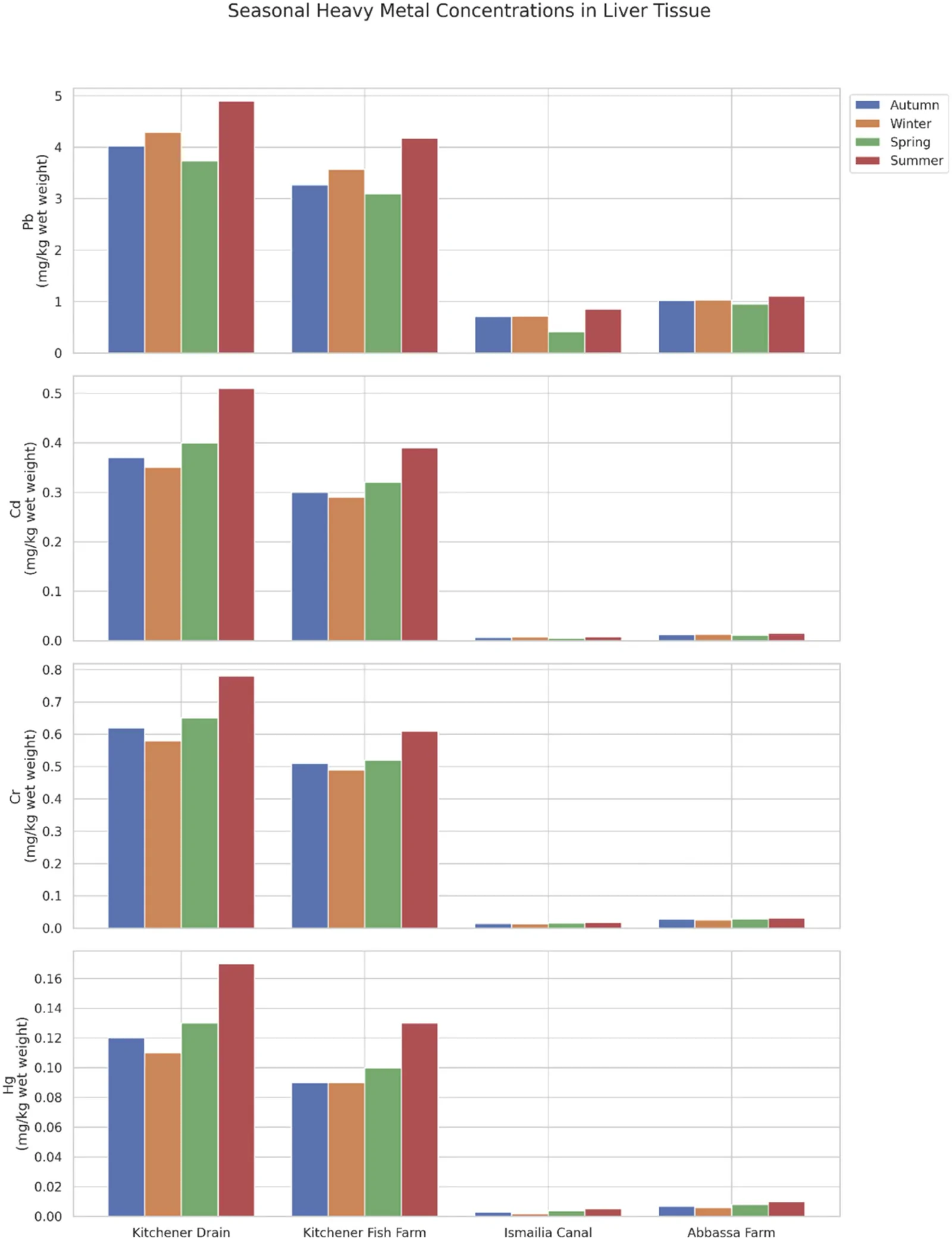
Heavy metal concentrations (Pb, Cd, Cr, Hg) in liver tissue of Nile tilapia during summer. Values are mean ± SD (n = 10 per site). Site differences were significant for all metals (p < 0.001).

Heavy metal concentrations in liver tissues of *Oreochromis niloticus* differed significantly across study sites (*p* < 0.001). The highest levels were detected in fish from the Kitchener Drain (mean: 4.24 ± 0.53 mg/kg). followed by the Kitchener Fish Farm (3.53 ± 0.47 mg/kg). Abbassa Farm (1.03 ± 0.22 mg/kg). and the Ismailia Canal (0.67 ± 0.19 mg/kg) (Table 4). Heavy metal accumulation in liver tissue during the summer peak contamination period is shown in Fig. 8.

**Table 4:** Seasonal heavy metal concentrations (mg/kg wet weight) in liver tissue of Nile tilapia (Oreochromis niloticus). Values are mean ± SD (n = 10 per site per season). Two-way ANOVA results: Site effect p < 0.001, Season effect p < 0.01.

Season	Site	Pb	Cd	Cr	Hg
Autumn	Kitchener Drain	4.02 ± 0.50	0.370 ± 0.071	0.620 ± 0.087	0.120 ± 0.026
	Kitchener Fish Farm	3.27 ± 0.47	0.300 ± 0.045	0.510 ± 0.053	0.090 ± 0.019
	Ismailia Canal	0.71 ± 0.19	0.006 ± 0.001	0.015 ± 0.002	0.003 ± 0.001
	Abbassa Farm	1.02 ± 0.07	0.012 ± 0.002	0.028 ± 0.002	0.007 ± 0.002
Winter	Kitchener Drain	4.29 ± 0.50	0.350 ± 0.071	0.580 ± 0.087	0.110 ± 0.026
	Kitchener Fish Farm	3.57 ± 0.47	0.290 ± 0.045	0.490 ± 0.053	0.090 ± 0.019
	Ismailia Canal	0.72 ± 0.19	0.007 ± 0.001	0.013 ± 0.002	0.002 ± 0.001
	Abbassa Farm	1.03 ± 0.07	0.013 ± 0.002	0.026 ± 0.002	0.006 ± 0.002
Spring	Kitchener Drain	3.74 ± 0.50	0.400 ± 0.071	0.650 ± 0.087	0.130 ± 0.026
	Kitchener Fish Farm	3.10 ± 0.47	0.320 ± 0.045	0.520 ± 0.053	0.100 ± 0.019
	Ismailia Canal	0.41 ± 0.19	0.005 ± 0.001	0.016 ± 0.002	0.004 ± 0.001
	Abbassa Farm	0.95 ± 0.07	0.011 ± 0.002	0.029 ± 0.002	0.008 ± 0.002
Summer	Kitchener Drain	4.90 ± 0.50	0.510 ± 0.071	0.780 ± 0.087	0.170 ± 0.026
	Kitchener Fish Farm	4.18 ± 0.47	0.390 ± 0.045	0.610 ± 0.053	0.130 ± 0.019
	Ismailia Canal	0.86 ± 0.19	0.008 ± 0.001	0.018 ± 0.002	0.005 ± 0.001
	Abbassa Farm	1.11 ± 0.07	0.015 ± 0.002	0.031 ± 0.002	0.010 ± 0.002

#### 3.3.2 Kidney tissue

**Fig. 9:**
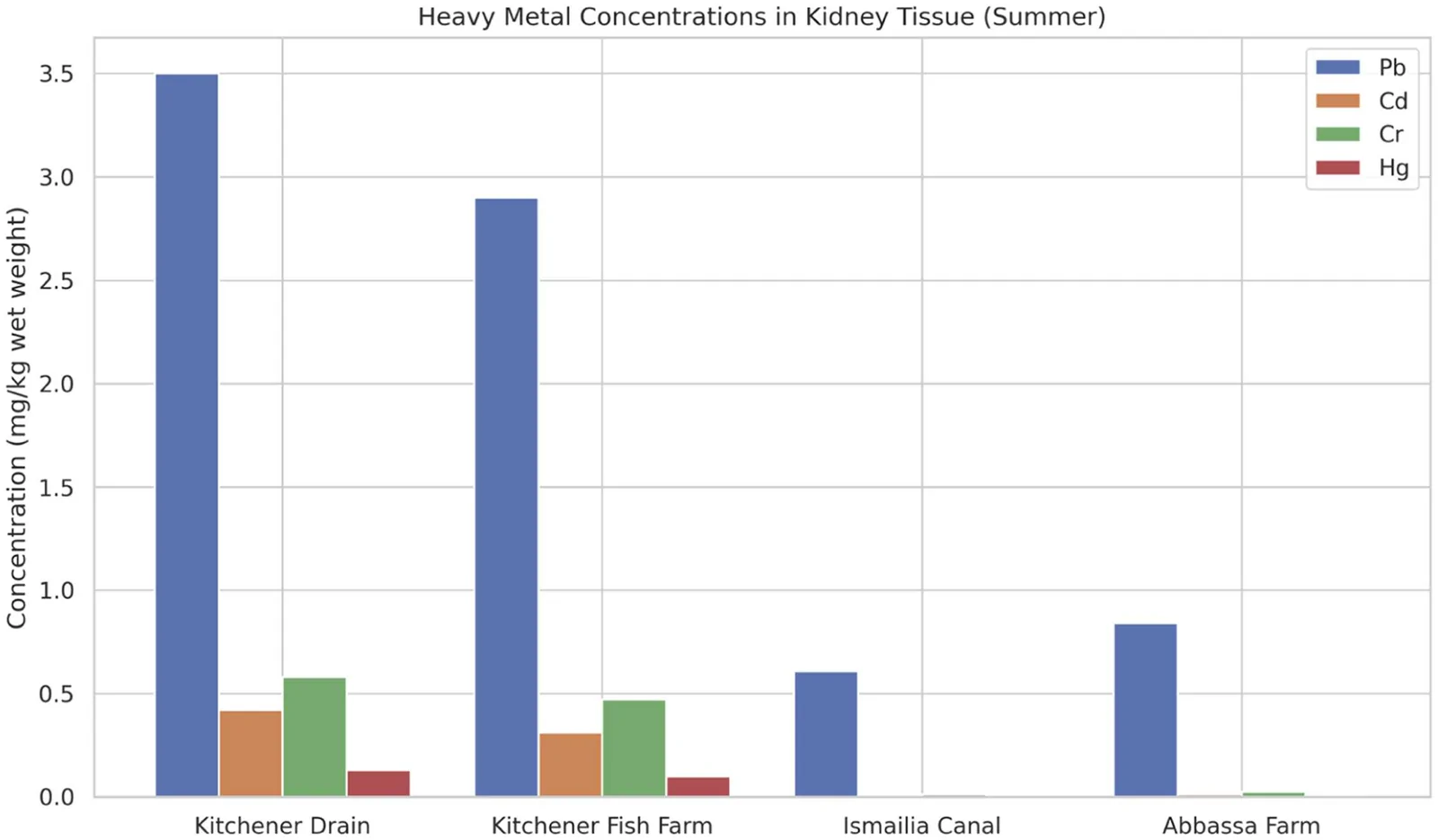
Heavy metal concentrations (Pb, Cd, Cr, Hg) in kidney tissue of Nile tilapia during summer. Values are mean ± SD (n = 10 per site).

Similar patterns were observed for metal concentrations in kidney tissue. with significantly higher levels in fish from the Kitchener sites compared to freshwater sites. Seasonal variations followed similar trends as observed in liver tissue. with peak values in summer. The full dataset for kidney metal accumulation during the summer peak contamination period is presented in Table 5. The corresponding metal concentrations in kidney tissue during summer are presented in Fig. 9.

**Table 5:** Average concentrations of heavy metals (mg/kg wet weight) in the kidney tissue of Nile tilapia from four sites during summer (peak contamination period). Values are shown as mean ± SD (n = 10 per site).

Site	Pb (mg/kg)	Cd (mg/kg)	Cr (mg/kg)	Hg (mg/kg)
Kitchener Drain	3.50 ± 0.45	0.42 ± 0.06	0.58 ± 0.07	0.13 ± 0.02
Kitchener Fish Farm	2.90 ± 0.40	0.31 ± 0.04	0.47 ± 0.05	0.10 ± 0.015
Ismailia Canal	0.61 ± 0.15	0.006 ± 0.001	0.014 ± 0.002	0.004 ± 0.001
Abbassa Farm	0.84 ± 0.06	0.012 ± 0.002	0.024 ± 0.002	0.008 ± 0.001

#### 3.3.3 Muscle tissue

**Fig. 10:**
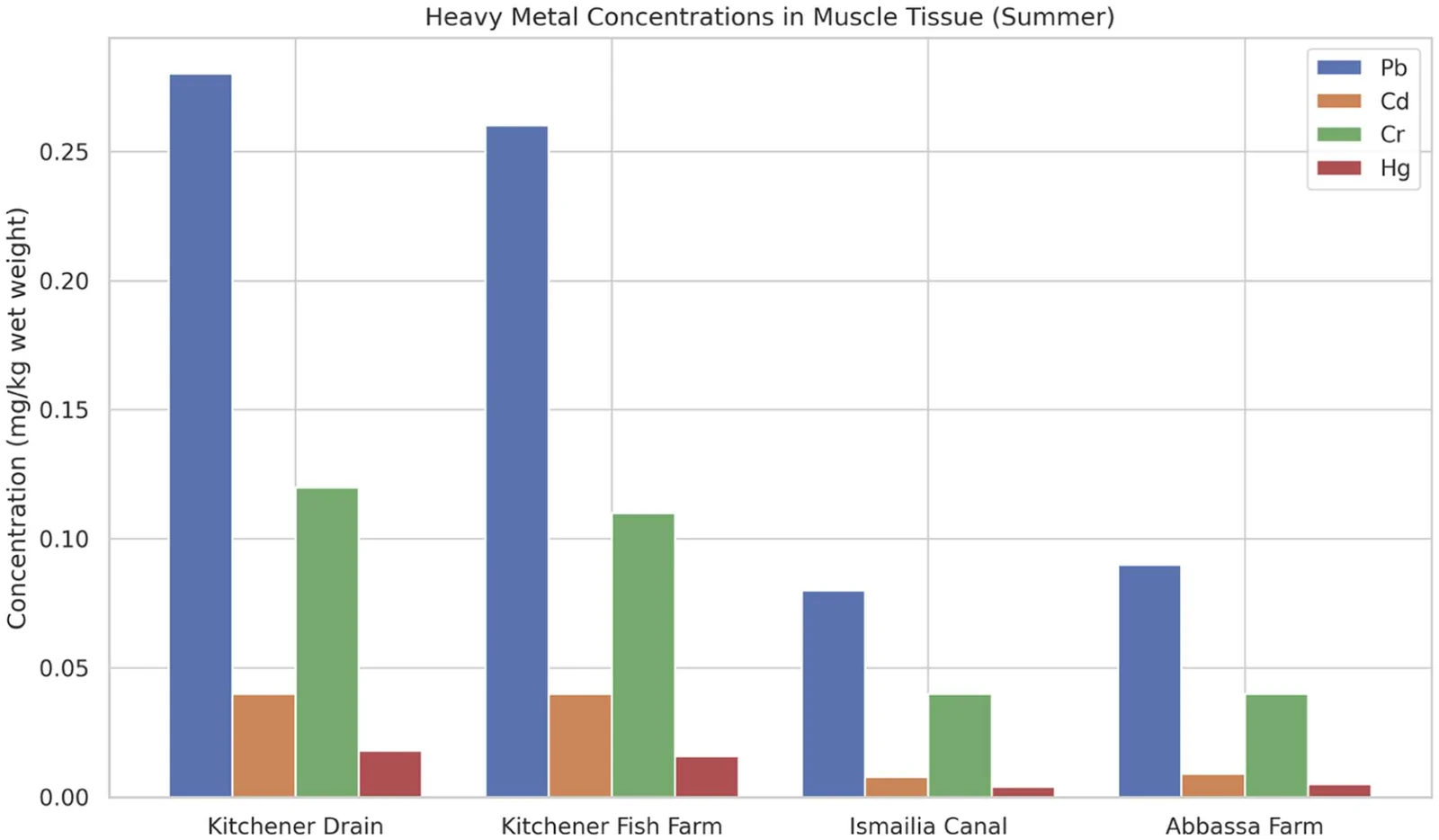
Heavy metal concentrations (Pb, Cd, Cr, Hg) in muscle tissue of Nile tilapia during summer compared to FAO/WHO permissible limits. The dashed red line indicates the FAO/WHO limit for Pb (0.3 mg/kg). All values remained below safety thresholds.

The levels of heavy metals in the muscle tissue of Oreochromis niloticus followed this order: Pb > Cd > Cr > Hg. Although elevated metal levels were detected in the liver and kidney tissues—especially in fish from the Kitchener Drain and Kitchener Fish Farm—the concentrations in muscle tissue remained below the FAO/WHO maximum permissible limits for human consumption at all locations (Table 6). *A comparison of muscle metal levels with FAO/WHO permissible limits during summer are depicted in Fig. 10. *

**Table 6:** Average concentrations of heavy metals (mg/kg wet weight) in the muscle tissue of Nile tilapia at four sites during summer (the peak contamination period). Values are presented as mean ± SD (n = 10 per site). FAO/WHO permissible limits are included for comparison.

Metal	Kitchener Drain	Kitchener Fish Farm	Ismailia Canal	Abbassa Farm	FAO/WHO Limit
Pb	0.28 ± 0.04	0.26 ± 0.04	0.08 ± 0.01	0.09 ± 0.01	0.3
Cd	0.04 ± 0.01	0.04 ± 0.01	0.008 ± 0.002	0.009 ± 0.002	0.05
Cr	0.12 ± 0.02	0.11 ± 0.02	0.04 ± 0.01	0.04 ± 0.01	0.15
Hg	0.018 ± 0.003	0.016 ± 0.003	0.004 ± 0.001	0.005 ± 0.001	0.5

### 3.4 Bioaccumulation factors (BAF)

**Fig. 11:**
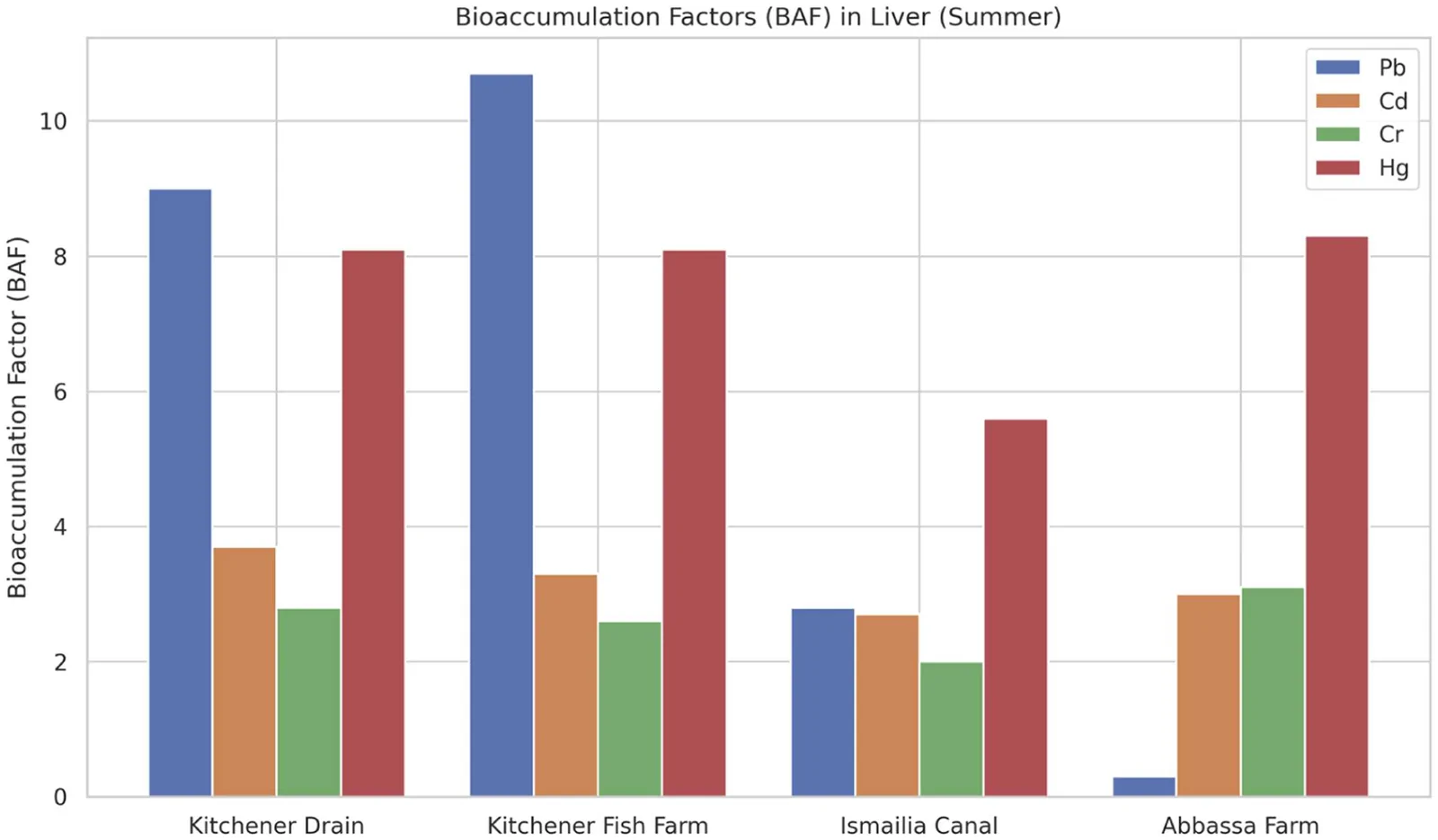
Bioaccumulation factors (BAF = C_tissue / C_water) for target metals in liver tissue of Nile tilapia during summer.

**Fig. 12:**
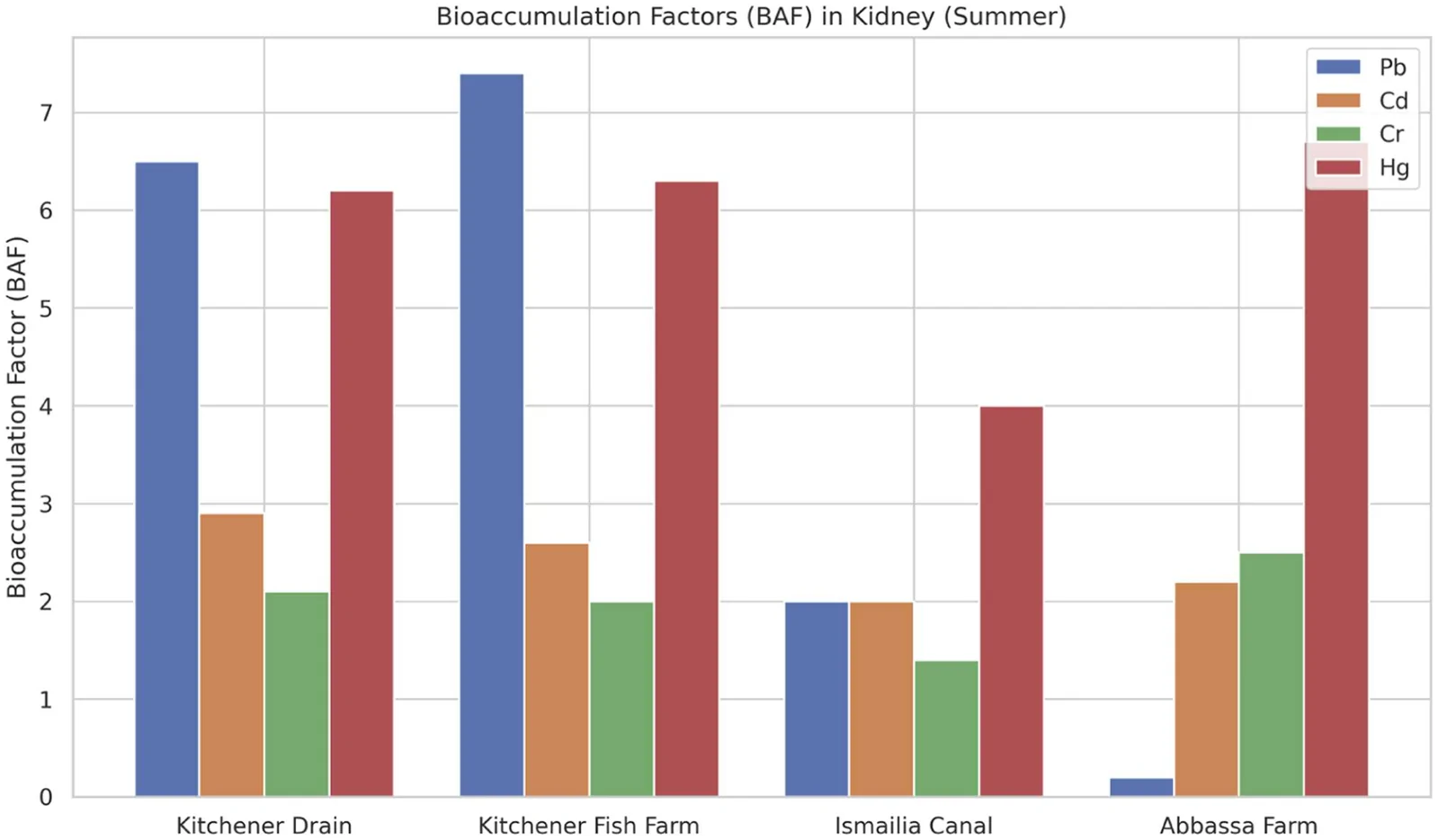
Bioaccumulation factors (BAF = C_tissue / C_water) for target metals in kidney tissue of Nile tilapia during summer.

**Fig. 13:**
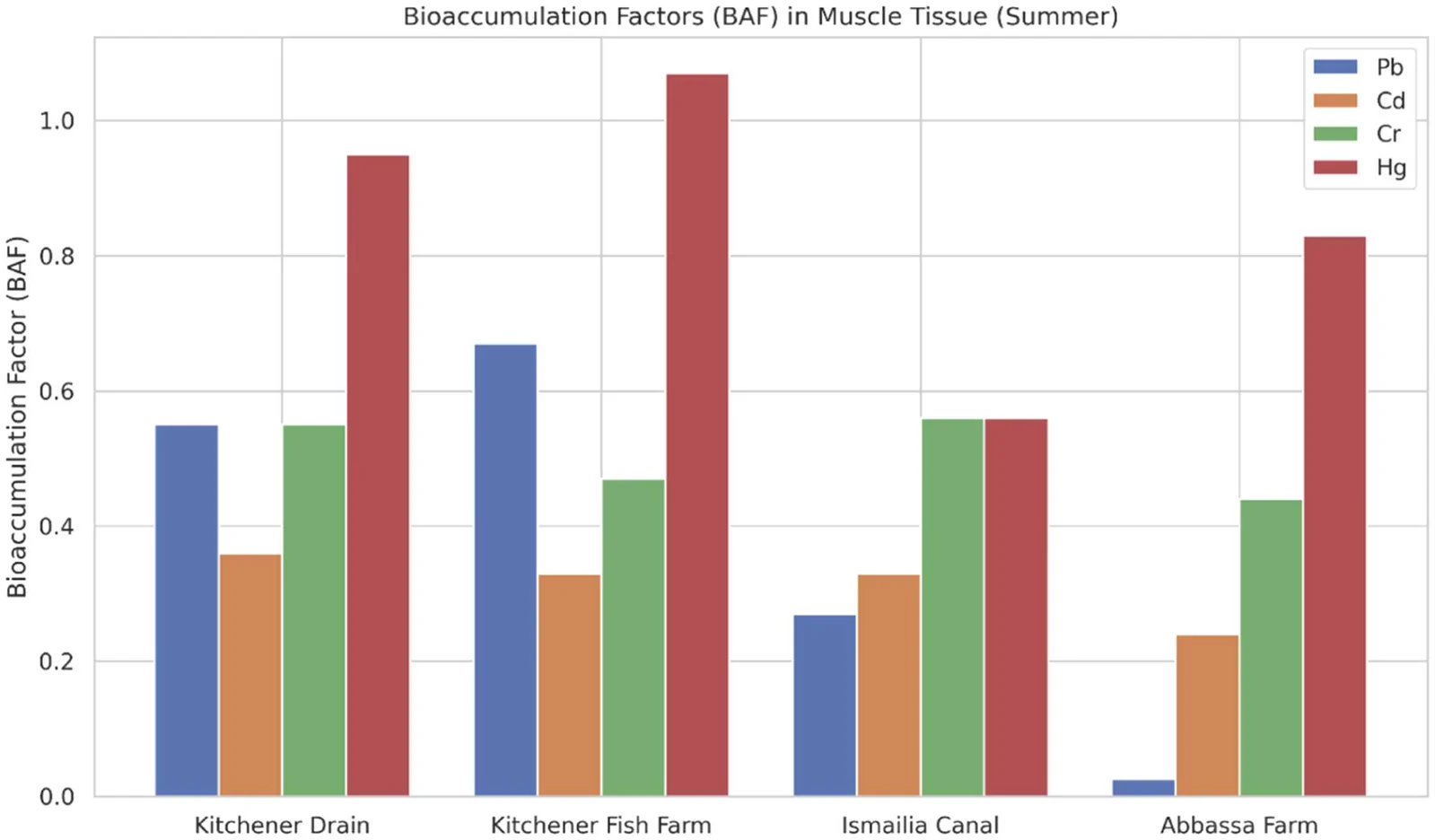
Bioaccumulation factors (BAF = C_tissue / C_water) for target metals in muscle tissue of Nile tilapia during summer.

BAF analysis revealed tissue-specific differences in metal accumulation. Interestingly, BAF values were highest at the Abbassa and Ismailia sites, despite lower environmental concentrations (Table 7). The bioaccumulation factors (BAF) for liver tissue during summer are displayed in Fig. 11**.** Kidney tissue BAF values for the same period are shown in Fig. 12**.** Muscle tissue BAF values during summer are presented in Fig. 13**.**

**Table 7:** Bioaccumulation factors (BAF = C_tissue / C_water) for target metals in different tissues of Nile tilapia during summer.

Tissue	Site	Pb	Cd	Cr	Hg
Liver	Kitchener Drain	9.0	3.7	2.8	8.1
	Kitchener Fish Farm	10.7	3.3	2.6	8.1
	Ismailia Canal	2.87	2.7	2.0	5.6
	Abbassa Farm	0.32	3.0	3.1	8.3
Kidney	Kitchener Drain	6.5	2.9	2.1	6.2
	Kitchener Fish Farm	7.4	2.6	2.0	6.3
	Ismailia Canal	2.03	2.0	1.4	4.0
	Abbassa Farm	0.24	2.2	2.5	6.7
Muscle	Kitchener Drain	0.55	0.36	0.55	0.95
	Kitchener Fish Farm	0.67	0.33	0.47	1.0
	Ismailia Canal	0.27	0.33	0.56	0.56
	Abbassa Farm	0.026	0.24	0.44	0.83

### 3.5 Correlation between water and tissue concentrations

Significant positive correlations were found between metal concentrations in water and corresponding concentrations in liver tissue (Pearson correlation: Pb: r = 0.89, p < 0.001; Cd: r = 0.91, p < 0.001; Cr: r = 0.86, p < 0.001; Hg: r = 0.82, p < 0.001). confirming that water source quality directly determines bioaccumulation risk

### 3.6 Human health domain: dietary exposure and risk assessment

Significant positive correlations were found between metal concentrations in water and corresponding concentrations in liver tissue (Pearson correlation: Pb: r = 0.89, p < 0.001; Cd: r = 0.91, p < 0.001; Cr: r = 0.86, p < 0.001; Hg: r = 0.82, p < 0.001), confirming that water source quality directly determines bioaccumulation risk. The Target Hazard Quotients (THQ) for individual heavy metals across all sites are shown in Table 8.

**Table 8:** Target Hazard Quotient (THQ) for individual metals.

Metal	Kitchener Drain	Kitchener Fish Farm	Ismailia Canal	Abbassa Farm
Pb	0.13	0.12	0.04	0.04
Cd	0.066	0.066	0.013	0.015
Cr	0.067	0.060	0.022	0.022
Hg	0.10	0.087	0.022	0.027

The seasonal and annual Hazard Index (HI) values for all four sites are provided in Table 9**.**

**Table 9:** Hazard Index (HI) by site and season.

Season	Kitchener Drain	Kitchener Fish Farm	Ismailia Canal	Abbassa Farm
Autumn	0.28	0.25	0.08	0.09
Winter	0.24	0.22	0.07	0.08
Spring	0.31	0.28	0.09	0.10
Summer	**0.36**	**0.33**	**0.10**	**0.10**
**Annual Mean**	**0.30**	**0.27**	**0.085**	**0.092**

**Fig. 14:**
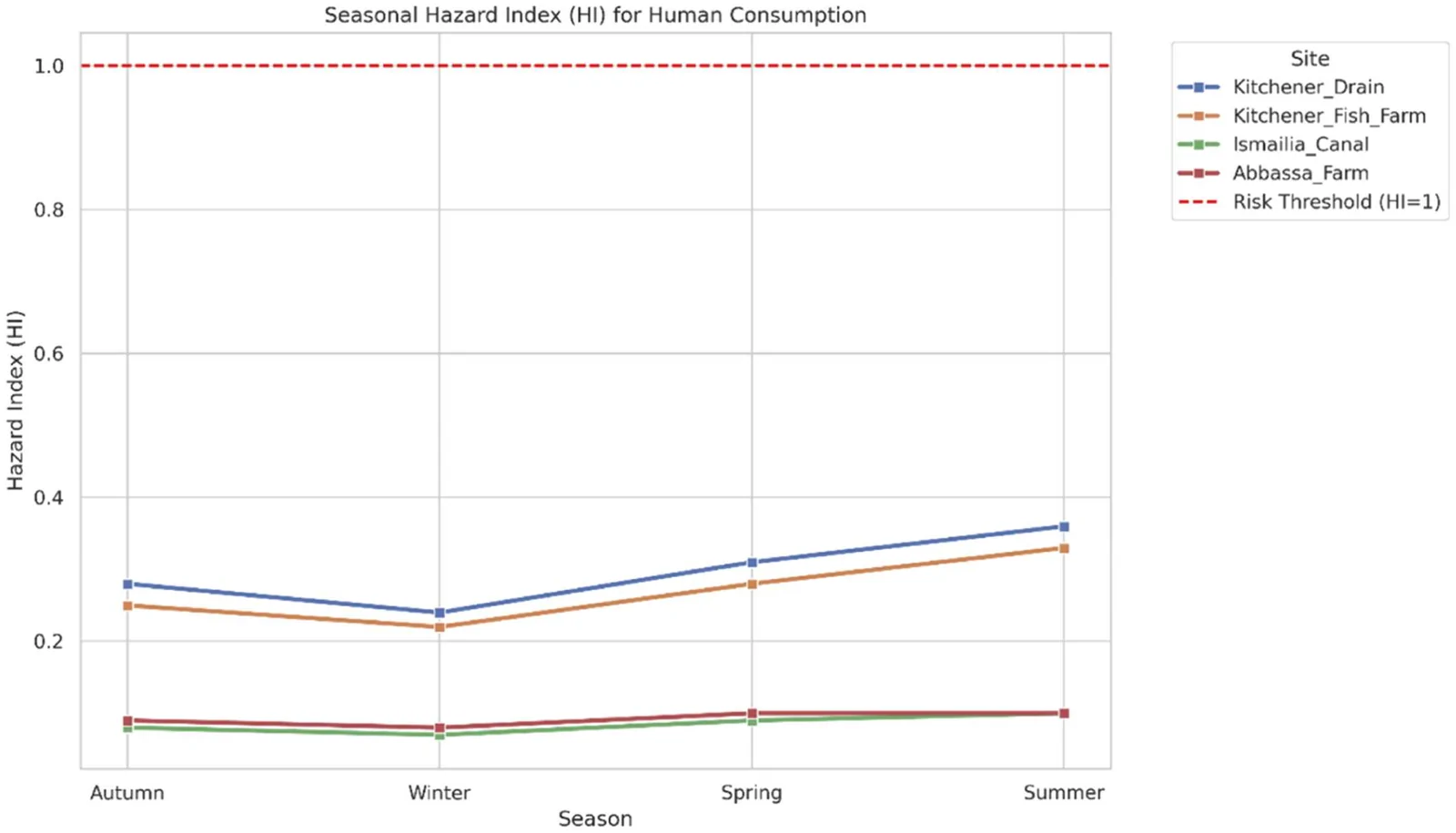
Seasonal variation in Hazard Index (HI) for heavy metal exposure through fish consumption. Values indicate the cumulative risk from Pb, Cd, Cr, and Hg. All HI values were below 1, indicating no acute health risk. but Kitchener Drain exhibited a 3.6-fold higher HI than reference sites. with peak values occurring in summer.

A comparison of the HI values obtained in this study with those reported in other international studies is given in Table 10*.* The seasonal variation in the cumulative Hazard Index (HI) for all sites is shown in Fig. 14**.**

**Table 10:** Comparison of Hazard Index (HI) values with international studies.

Location	HI (range)	Reference
Kitchener Drain (this study)	0.24–0.36	-
Nile Delta. Egypt (other studies)	0.15–0.45	[31]
Lake Manzala. Egypt	0.20–0.50	[32]
Mediterranean Sea	0.10–0.30	[33]
Global average	< 1.0	US EPA. 2021

### 3.7 One health integration: correlations across domains

.

## 4 Discussion

### 4.1 One health integration: a novel approach for egyptian aquaculture

This study offers the first comprehensive One Health evaluation of heavy metal pollution in Egyptian aquaculture, combining environmental monitoring, fish physiology, and human health risk assessment. The strong correlations observed across all three domains (r > 0.85, p < 0.001) provide empirical support for the One Health framework, demonstrating that environmental pollution directly affects animal health and indirectly influences human consumers. This integrated approach is particularly relevant for water-scarce regions like Egypt, where the reuse of agricultural drainage water in aquaculture creates complex health risks that transcend traditional disciplinary boundaries.

### 4.2 Environmental health domain: the kitchener drain as a pollution hotspot

Our findings confirm that the Kitchener Drain is a major pollution hotspot, with heavy metal concentrations far exceeding WHO guidelines, especially during summer. Lead levels (0.54 mg/L) surpassed WHO limits by 54-fold, cadmium (0.14 mg/L) by 28-fold, chromium (0.28 mg/L) by 5.6-fold, and mercury (0.021 mg/L) by 21-fold. These elevated levels reflect the cumulative impact of untreated industrial effluents, agricultural runoff, and domestic wastewater entering the drainage system [9], [22]. The seasonal pattern, with peak concentrations in summer, can be attributed to reduced water flow, increased evaporation concentrating pollutants, and intensified agricultural activities during the growing season [21]. This seasonal variation has important implications for both fish health and human exposure, as summer represents a critical risk period that requires intensified monitoring and management.

### 4.3 Animal health domain: fish as sentinels of environmental contamination

#### 4.3.1 Bioaccumulation patterns

Fish from Kitchener sites showed significant metal accumulation in liver tissues, with Pb reaching 4.90 mg/kg, supporting the liver's role as the primary detoxification organ [34], [35]. The bioaccumulation pattern (liver > kidney > muscle) aligns with previous studies [24], [36] and reflects the physiological roles of these organs in detoxification, filtration, and storage. Critically, muscle tissue, which represents the edible portion for human consumption, remained within FAO/WHO limits at all sites, indicating that current consumption likely does not pose immediate health risks. However, fish from Kitchener Drain approached the Pb limit (0.28 mg/kg vs. 0.3 mg/kg), suggesting that safety margins are limited and that even minor increases in contamination could compromise food safety. Temperature and water exchange are known to influence water quality in aquaculture ponds [37], while the mechanisms of environmentally induced oxidative stress in aquatic animals have been extensively reviewed [38].

### 4.4 Human health domain: current safety and future concerns

#### 4.4.1 Risk assessment: current status

All THQ values were below 1, and all HI values were below 1, indicating no significant non-carcinogenic health risk from current fish consumption patterns. This finding is reassuring for consumers and regulatory authorities. However, several factors warrant careful consideration. First, the narrow safety margins for Pb at Kitchener Drain (0.28 mg/kg) are just 7% below the FAO/WHO limit of 0.3 mg/kg, meaning that small increases in contamination could cause levels to exceed the limit. Second, the cumulative effects, as reflected by the Hazard Index (HI = 0.36 at Kitchener Drain), are 3.6-fold higher than at reference sites (HI = 0.10), indicating that even within safe limits, the overall burden is significantly greater for consumers of fish from contaminated areas. Third, seasonal peaks in summer, where HI values are 50% higher than in winter, suggest that consumers who eat more fish during summer may face increased exposure.

#### 4.4.2 Comparison with international standards

Our HI values (0.24–0.36) are comparable to those reported in other studies in the Nile Delta region (0.15–0.45; [31], [32]) and are below global safety thresholds. However, the consistent elevation at Kitchener sites compared to reference sites demonstrates the tangible impact of drainage water use on consumer exposure. This underscores the need for region-specific risk assessments that account for local consumption patterns and contamination sources.

#### 4.4.3 Vulnerable populations

Our risk assessment used average consumption rates (115 g/day) and body weight (70 kg) for the general population. However, certain subgroups may face increased risks. Fishermen and their families may consume up to 2–3 times more fish than the general population, while children have lower body weight, resulting in higher EDI per kg body weight. Pregnant women are also at greater risk, as the neurotoxic effects of Pb and Hg are particularly concerning during fetal development. Future research should perform stratified risk evaluations for these vulnerable groups. Bacterial indicators of water quality in Egyptian fish farms have also been discussed as complementary tools for assessing environmental health risks [39].

### 4.5 One health integration: key findings

The strong correlations across domains offer empirical backing for the One Health framework. These findings demonstrate that fish health and human health are inextricably linked to environmental quality, the core principle of One Health. Specifically, Pb concentrations in water showed a strong positive correlation with Pb in muscle (r = 0.89), confirming that environmental contamination directly determines fish contamination. Similarly, Pb in muscle correlated strongly with THQ (r = 0.95), demonstrating that fish contamination determines human exposure risk.

### 4.6 Seasonal dynamics and risk management

The consistent seasonal pattern (summer > spring > autumn > winter) across all domains has important implications for risk management. Water Pb levels were 28% higher in summer compared to winter, reflecting increased irrigation runoff. Muscle Pb levels were 12% higher, indicating higher uptake at warmer temperatures. The HI was 50% higher in summer, corresponding to higher consumer exposure. These findings suggest that monitoring and mitigation efforts should be intensified during spring and summer, when risks are highest.

### 4.7 Policy recommendations within a one health framework

Based on our findings, we propose the following integrated recommendations across environmental, animal, and human health domains. In the environmental health domain, we recommend strengthening enforcement of industrial discharge limits into the Kitchener Drain, implementing low-cost treatment options such as sedimentation ponds and constructed wetlands for drainage water before aquaculture use, increasing water exchange rates during summer to dilute contaminants, and establishing quarterly water quality monitoring programs at all aquaculture sites using drainage water. In the animal health domain, we recommend developing fish health standards based on physiological responses rather than tissue residues alone, considering antioxidant-supplemented feeds for fish in contaminated systems to support detoxification, and reducing stocking densities during high-risk seasons to minimise stress. In the human health domain, we recommend informing consumers about seasonal variations in risk and recommending consumption patterns, implementing labelling of fish origin to allow informed consumer choices, developing specific consumption advice for pregnant women and children, and expanding regular testing of marketed fish for heavy metals. Finally, integrated One Health recommendations include establishing a joint committee between the Ministry of Environment, the Ministry of Agriculture, and the Ministry of Health for coordinated action, developing an integrated database linking environmental, animal, and human health data, funding longitudinal studies on chronic effects and intervention effectiveness, and training professionals across sectors in One Health approaches.

### 4.8 Study limitations and future research

#### 4.8.1 Limitations

This study provides comprehensive data but acknowledges certain limitations. The sampling covered a one-year cycle; multi-year studies would clarify inter-annual variability in metal concentrations and biological responses. The analysis was limited to four heavy metals; interactions with other contaminants (e.g., pesticides, pharmaceuticals, microplastics) warrant further investigation. While biochemical markers indicated organ damage, histopathological confirmation of tissue alterations was not performed. Gene expression analysis was focused solely on metallothionein; expanding molecular analysis to include other stress and immune-related genes (e.g., HSP70, IL-1β, TNF-α) would provide a more complete picture of the molecular response. Additionally, the absence of human biomarker data (hair, blood, urine) limits the direct assessment of human exposure, and the risk assessment used population averages rather than stratifying by age, gender, or consumption patterns.

#### 4.8.2 Future research directions

In the short term (1–2 years), we recommend human biomonitoring of fishermen and their families in the Kitchener region, analysis of additional contaminants (pesticides, PCBs, pharmaceuticals), and seasonal risk communication studies. In the medium term (2–5 years), we suggest intervention studies to evaluate water treatment effectiveness, longitudinal cohort studies tracking fish consumers' health outcomes, and economic analysis of One Health interventions. In the long term (5–10 years), we advocate for the development of a national One Health surveillance system for aquaculture, policy impact assessment, and regional collaboration with other water-scarce countries.

## 5 Conclusions

This study offers the first comprehensive One Health evaluation of heavy metal contamination in Egyptian aquaculture, combining environmental monitoring, fish physiology, and human health risk assessment. The main conclusions are:

### 5.1 Environmental health

The Kitchener Drain is heavily contaminated with heavy metals, with summer concentrations exceeding WHO guidelines by 5–54 times. The Ismailia Canal and Abbassa Farm exhibit considerably better water quality, confirming that the choice of water source is the main factor influencing contamination risk.

### 5.2 Animal health

Fish from Kitchener sites show significant metal bioaccumulation in liver tissues (Pb: 4.90 mg/kg, Cd: 0.51 mg/kg) and notable physiological stress responses.

• Upregulated metallothionein gene expression (up to 5.1-fold). confirming a molecular response.

Crucially, fish muscle tissue—the edible part—remains within FAO/WHO limits at all sites, indicating no immediate food safety violation. However, the physiological stress observed in fish serves as an early warning of potential human health risks.

### 5.3 Human health

Current risk assessment shows:

• All THQ values are below 1 (indicating no significant risk from individual metals).
• All HI values are below 1, indicating there is no significant cumulative risk.
• However, fish from Kitchener Drain have HI values 3.6 times higher than those at reference sites.
• Summer poses the highest risk with an HI of 0.36. which is 50% higher than in winter.

### 5.4 One health integration

Strong correlations across domains validate the One Health approach:

• Environmental contamination → Fish contamination (r = 0.89)
• Fish contamination → Fish physiological stress (r = 0.82)
• Fish contamination → Human health risk (r = 0.95)
• Fish biomarkers → Human health risk (r = 0.88)

### 5.5 Overall assessment

Although current heavy metal levels in edible fish muscle are not an immediate health risk, the high HI values and signs of physiological stress in fish suggest possible long-term problems. The limited safety margins (Pb at Kitchener: 0.28 mg/kg versus the limit of 0.3 mg/kg) imply that even slight increases in contamination could push levels above regulatory limits.

The One Health framework shows that environmental degradation directly affects animal health and, indirectly, human consumers. This integrated approach provides a scientific foundation for coordinated efforts across the environmental, agricultural, and health sectors.

## Ethical Approval

The study was conducted in accordance with the guidelines of the Central Laboratory for Aquaculture Research (CLAR), Abbassa, Egypt. The experimental protocol involving Nile tilapia (Oreochromis niloticus) was approved by CLAR's Institutional Animal Care and Use Committee. All efforts were made to minimise animal suffering and to reduce the number of fish used.

## Funding

No specific funding was received for this study.

## CRediT authorship contribution statement

**Mohamed A. Al-Zahaby:** Writing – review & editing, Resources, Investigation. **Alam Eldeen Farouk:** Writing – review & editing, Resources, Investigation. **Mohamed Farahat Abdelmawla:** Writing – original draft, Visualization, Project administration, Methodology, Investigation, Formal analysis, Conceptualization. **Ibrahim Shaker:** Writing – review & editing, Validation, Supervision. **Ahmed M.D. Elshafei:** Writing – review & editing, Resources, Investigation.

## Declaration of Competing Interest

The authors declare that they have no known competing financial interests or personal relationships that could have appeared to influence the work reported in this paper.

## Data Availability

Data will be made available on request.
